# Cell-Level Analysis Visualizing Photodynamic Therapy with Porphylipoprotein and Talaporphyrin Sodium

**DOI:** 10.3390/ijms232113140

**Published:** 2022-10-28

**Authors:** Mayuka Kamiyanagi, Atsushi Taninaka, Shunta Ugajin, Yu Nagoshi, Hiromi Kurokawa, Takahiro Ochiai, Yusuke Arashida, Osamu Takeuchi, Hirofumi Matsui, Hidemi Shigekawa

**Affiliations:** 1 Faculty of Pure and Applied Sciences, University of Tsukuba, 1-1-1 Tennodai, Tsukuba 305-8573, Japan; 2 TAKANO Co., Ltd., Miyada-mura, Kamiina-gun, Nagano 399-4301, Japan; 3 Fuculty of Medicine, University of Tsukuba, 1-1-1 Tennodai, Tsukuba 305-8575, Japan

**Keywords:** photodynamic therapy (PDT), talaporphyrin sodium (NPe6), porphylipoprotein (PLP), phagosome, atomic force microscopy, elasticity

## Abstract

We revealed the difference in the mechanism of photodynamic therapy (PDT) between two photosensitizers: porphylipoprotein (PLP), which has recently attracted attention for its potential to be highly effective in treating cancer, and talaporphyrin sodium (NPe6). (1) NPe6 accumulates in lysosomes, whereas PLP is incorporated into phagosomes formed by PLP injection. (2) PDT causes NPe6 to generate reactive oxygen species, thereby producing actin filaments and stress fibers. In the case of PLP, however, reactive oxygen species generated by PDT remain in the phagosomes until the phagosomal membrane is destroyed, which delays the initiation of RhoA activation and RhoA*/ROCK generation. (4) After the disruption of the phagosomal membrane, however, the outflow of various reactive oxygen species accelerates the production of actin filaments and stress fibers, and blebbing occurs earlier than in the case of NPe6. (5) PLP increases the elastic modulus of cells without RhoA activity in the early stage. This is because phagosomes are involved in polymerizing actin filaments and pseudopodia formation. Considering the high selectivity and uptake of PLP into cancer cells, a larger effect with PDT can be expected by skillfully combining the newly discovered characteristics, such as the appearance of a strong effect at an early stage.

## 1. Introduction

Photodynamic therapy (PDT) treats diseases by injecting photosensitizers into the body and irradiating them with light to generate reactive oxygen species (ROS) [1,2,3,4]. It is noninvasive with few adverse effects and exhibits a high therapeutic effect on lesion areas that are difficult to treat with surgical techniques. It is used not only for cancer therapy but also for conditions such as age-related macular degeneration and skin diseases [5,6,7]. Recently, as a method to efficiently inactivate pathogens without inducing resistant bacteria, antimicrobial photodynamic therapy has been developed and applied to treatment for periodontal disease [8,9,10,11]. Porphyrin has been mainly used as a photosensitizer. In PDT, porphyrin ring is photoexcited to generate singlet oxygen, which is converted into OH radicals and other ROS, such as H_2_O_2_, which destroy the cytoplasm [1,3,12,13]. The performance of the photosensitizer determines the therapeutic effect of PDT, for example, the degree of selective uptake into the affected area, permeability, and persistence. Photosensitizers accumulate in mitochondria, endoplasmic reticulum, and lysosomes at the cellular level. That changes the persistence of photosensitizers and the types of organelles destroyed by ROS. For example, there is a difference in the effect of PDT with Porfimer sodium and Talaporfin sodium (NPe6). Therefore, various photosensitizers suitable for treatment have been developed so far [13].

On the other hand, advances in technology have made drug delivery practical. Research is being actively pursued for further development. Although attached polyethylene glycol polymer chains (PEG) to molecules or nanoparticles have been clinically approved, there remain several concerns including immunogenicity, anti-PEG immune response, biocompatibility and toxicity issues following chronic administration. Previous attempts to solve the issues typically involve developing PEG linker-removable approaches [14,15].

Porphylipoprotein (PLP) nanoplatform integrates a variety of imaging and therapeutic functionalities including positron emission tomography (PET) imaging, activatable near-infrared (NIR) fluorescence and photodynamic therapy (PDT) [14,15]. The tumor-selective activation of NIR fluorescence facilitated the delineation of tumor, the selectively activated photodynamic reactivity enabled potent PDT for tumor ablation and surgery bed cleaning [14,15].

Heme Carrier Protein 1 which is a transporter involved in low-affinity heme-Fe or folate (HCP1) is expressed more in cancer cells than in normal cells [16,17,18]. Therefore, molecules with a porphyrin ring passing through HCP1 have been considered for use as photosensitizers [18]. In fact, in vivo and in vitro, PLP was found to be selectively taken up by cancer cells in large amounts and have a large therapeutic effect [18]. A high effect has also been confirmed in NOZ (Human gallbladder carcinoma cell line) cells [18]. However, the mechanism for PDT has not been clarified yet. Therefore, in this study, we investigated the properties of PLP in detail by comparing them with those of NPe6.

In a previous paper, we focused on PDT using talaporphyrin sodium (NPe6) and investigated how it behaves in cancer cells by combining conventional techniques such as luminescence and Western blotting with atomic force microscopy (AFM) [19]. We examined the mechanism of actin filament formation by analyzing the change in elasticity of living cells. As a result, we succeeded in clarifying the relationship between stress fiber generation and blood flow disturbance, which was difficult to analyze by optical methods [19]. We used similar methods in this study. As a result, it became clear that the high function of PLP is caused by a mechanism involving phagosomes known in the field of autophagy. This function has the potential to enhance the effectiveness of PDT and is expected to play an important role in the future.

## 2. Results and Discussions

### 2.1. Subcellular Distribution of Injected NPe6 and PLP

**Figure 1:**
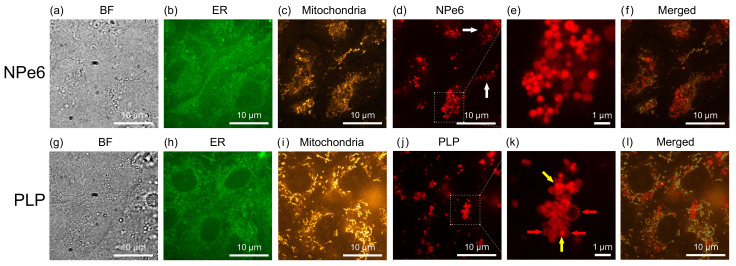
Confocal microscopy observation of RGK1 cells after NPe6 or PLP was added and allowed to stand for 24 h. (**a**,**g**) Bright field (BF) images, (**b**,**h**) endoplasmic reticulum (ER) fluorescence images, (**c**,**i**) mitochondria fluorescence images, (**d**,**j**) NPe6 and PLP fluorescence images and (**e**,**k**) their magnified views, and (**f**,**l**) merged images of (**c**,**d**), and (**i**,**j**), respectively. ER-Tracker™ Green (Invitrogen™) and MitoTracker™ Orange CMTMRos (Invitrogen™) were used for fluorescence staining of ER and mitochondria, respectively. After adding these fluorescent probes together in a sample with standing of 24 h after doping NPe6 or PLP, and leaving the mixture for 30 min, measurements were carried out while changing the excitation light and filters. Then, PDT was performed to observe the distributions of NPe6 and PLP. As shown in Figure 1e,k, NPe6 and PLP accumulated in granular substances with a diameter of about 200 nm–1 μm.

The cells used in this study were rat gastric mucosa-derived cancer-like mutant RGK1 cells [20]. NPe6 or PLP was added to RGK1 cells and confocal microscope observations were carried out 24 h later (see Materials and Methods for more details). Figure 1 shows results: (a,g) bright field (BF) images, (b,h) endoplasmic reticulum fluorescence (ER) images, (c,i) mitochondria fluorescence images, (d,j) magnified views of (e,k), and (f,l) merged images of the distributions of mitochondria and photosensitizer images. We first examined the intracellular distributions of NPe6 and PLP.

Figure 1e,k show that NPe6 and PLP accumulate in granular substances with a diameter of about 200 nm − 1 μm. Lysosomes and phagosomes are vesicles with a diameter of 200 nm − 1 μm among intracellular organelles. As can be seen from the merged figures, in the case of NPe6, NPe6 are distributed even in areas where no mitochondria are distributed, as indicated by the white arrows in Figure 1d. This is in good agreement with the fact that NPe6 is known to accumulate in lysosomes [21]. In addition, NPe6 shows uniform fluorescence inside the granular material in the enlarged view (Figure 1e). Therefore, NPe6 accumulated in the lysosomes, and PLP is considered to accumulate in phagosomes. Since water-soluble porphyrin derivatives accumulate in lysosomes [21,22], NPe6 is considered to be dissolved in the fluid inside lysosomes.

On the other hand, in the case of PLP, the granular substances aggregate near mitochondria, and many of them have a ring shape, as expected and indicated by the red arrows in Figure 1k. Phagosomes are formed at the ER and the mitochondria contact sites and have a circular shape [23]. Therefore, PLP is considered to accumulate in the circular membrane of phagosomes. Since phagosomes and lysosomes (indicated by the yellow arrows in Figure 1k) are known to fuse to form phagolysosomes [24,25,26,27,28,29,30,31], the bright spots with diameter of several hundred nanometers in Figure 1k may be lysosomes. The relationship between PLP, phagosomes, and lysosomes will be examined later.

### 2.2. Time-Lapse Observation by Phase-Contrast Microscopy

In general, when the light intensity of PDT was high (300 mW/cm^2^), cells swelled and then ruptured (necrosis), and when the light intensity was low (100 mW/cm^2^), blebbing occurred in about 10 min [19]. Furthermore, when PDT was performed at a lower light intensity as 34 mW/cm^2^, the blebbing was delayed, and the cell membrane hardened, causing obstruction of the blood flow and destruction of cancer cells [19].

In a previous study using NPe6, irradiation with light of 34 mW/cm^2^ at a wavelength of 655 nm for 1 min generated ROS, immediately after which actin filaments were generated, and, myosin light chain formation subsequently occurred after 5 min, leading to the formation of stress fibers [19,32]. Therefore, to compare the mechanism of stress fiber formation, RGK1 was first irradiated with light of 34 mW/cm^2^ at a wavelength of 655 nm for 1 min under the same sample conditions, that is, RGK1 that had been allowed to stand for 24 h after the addition of NPe6 or PLP, and time-lapse observation was performed with a phase-contrast fluorescence microscope.

**Figure 2:**
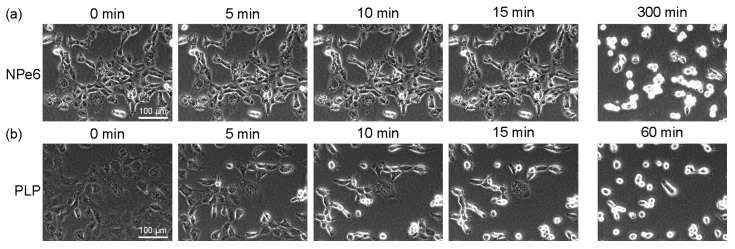
Time-lapse phase-contrast images of RGK1, which had been left standing in an incubator for 24 h after adding NPe6 (**a**) or PLP (**b**), then irradiated with light of 34 mW/cm^2^ at a wavelength of 655 nm for 1 min.

Figure 2a,b show phase-contrast observation images of NPe6 and PLP, respectively. Most of the cells to which NPe6 was added did not start blebbing (undergo a “death dance”) [33] even after 15 min, and some cells started to bleb from about 60 min. In contrast, cells to which PLP was added began to contract 5 min after 1 min light irradiation, and blebbing occurred 10–15 min after irradiation. In addition, the time required for almost all cells in the observed area to bleb was 300 min for NPe6 and 60 min for PLP. These results suggest that PLP induces excessive myosin light chain activation [33], which triggers blebbing, in a shorter time than NPe6.

In Figure 2, the amount of light is the same the both NPe6 and PLP-added samples. Thus, when the amount of light is increased, PLP is more effective in directly destroying cancer cells through necrosis or necroptosis. The fact that PLP is taken up by cancer cells in large amounts with high selectivity also enhances the therapeutic effect of this method.

### 2.3. Fluorescence Observation of Actin Filament Formation Process

When PDT is performed with a low light intensity, it has a therapeutic effect not by the immediate destruction of cancer cells but by the disturbance of blood flow [19,32,34,35]. The ROS generated by PDT trigger the formation of stress fibers through the activation of RhoA (Ras homolog family member A), producing actin filaments and myosin light chains [1,16,30]. In this case, the process of actin filament formation can be observed by fluorescence by staining actin filaments with SPY-555 (Cytoskeleton Inc., Denver, CO, USA) [36,37]. This a fluorescent probe enables the direct imaging of actin filaments in living cells [36,37].

**Figure 3:**
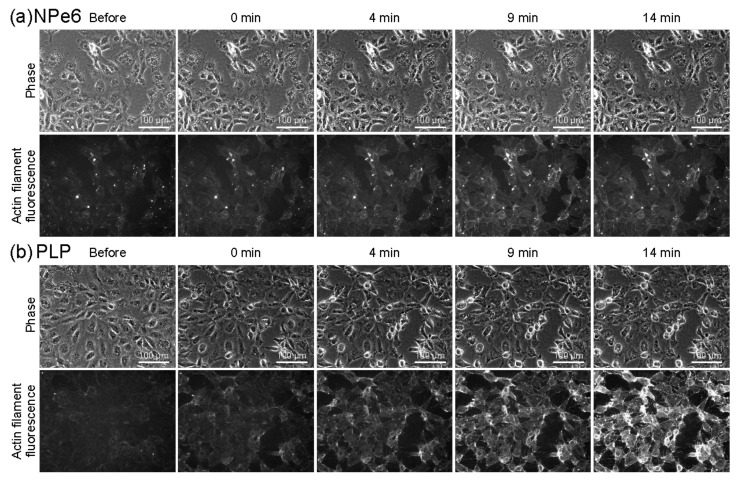
Results of time-lapse observation with a fluorescence microscopy. NPe6 (**a**) and PLP (**b**)-doped RGK1 samples were allowed to stand for 24 h in an incubator and then irradiated with a light of 34 mW/cm^2^ at a wavelength of 655 nm for 1 min.

Figure 3a,b, respectively show phase-contrast observation images and fluorescence observation images of actin filaments stained with SPY555 after PDT of RGK1 with NPe6 and PLP. For NPe6, as shown in Figure 2, the phase-contrast images showed almost no structural changes in most of the cells even 14 min after exposure to light for 1 min. The fluorescence intensity of actin filaments slightly increased immediately after light irradiation and gradually increased until 14 min after light irradiation. For PLP, it can be seen from the phase-contrast observation images that the cells began to contract 4 min after 1 min of light irradiation, and many cells had contracted by 14 min after light irradiation. The fluorescence intensity of actin filaments hardly increased immediately after light irradiation but started to increase after 4 min.

**Figure 4:**
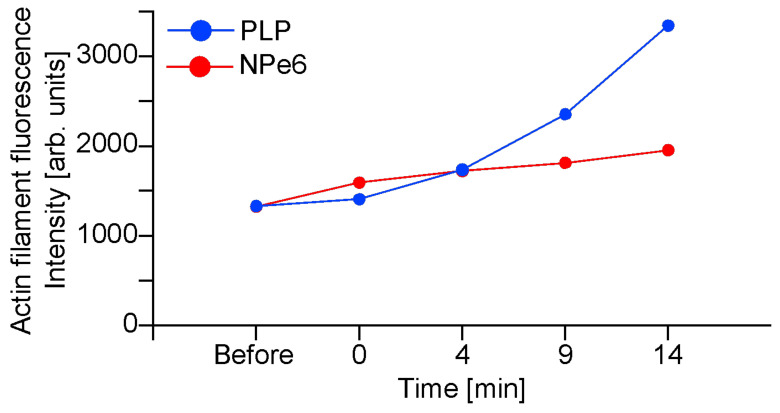
Results of quantifying the fluorescence intensity from the fluorescence observation images in Figure 3.

Figure 4 shows the results of quantifying the fluorescence intensity from the images in Figure 3. NPe6 showed an increase in fluorescence intensity after 1 min of irradiation. On the other hand, in PLP, the fluorescence intensity hardly changed after 1 min of irradiation, but the fluorescence intensity had increased 4 min after irradiation and reached the same level as that of NPe6. After 9 min, NPe6 continued to show a slight increase, while PLP showed a sharp increase in fluorescence intensity. This indicates that the production of actin filaments during 1 min of light irradiation is lower in PLP than in NPe6. The rapid increase in fluorescence intensity at 9 min for PLP is considered to be due to the influx of SPY-555 into cells due to damage to the cell membrane, as blebbing begins at 10–15 min according to Figure 2. This is because SPY-555 emits light when it enters the cells and interacted with actin filaments. In the case of NPe6, as shown in the previous paper, an increase similar to that of PLP was observed from about 30 min [19]. This is due to the time lag in which the cell membrane is damaged.

### 2.4. Analysis of RhoA Activation by Western Blotting

RhoA activation can be evaluated by Western blotting to assess RhoA*/ROCK binding and subsequent actin filament formation, where RhoA* is activated RhoA. As revealed in the previous NPe6 study, there is a threshold amount of ROS that is involved in the increase in actin filament production [19]. That is, there is an upper limit to the concentration of RhoA activated and binds to ROCK. In the fluorescence observation, no increasing in luminescence of PLP was observed immediately 1 min of light irradiation. Therefore, to determine whether RhoA activation and RhoA*/ROCK binding occurred in PLP-PDT as in NPe6, changes in the amount of RhoA were quantified by Western blotting.

**Figure 5:**
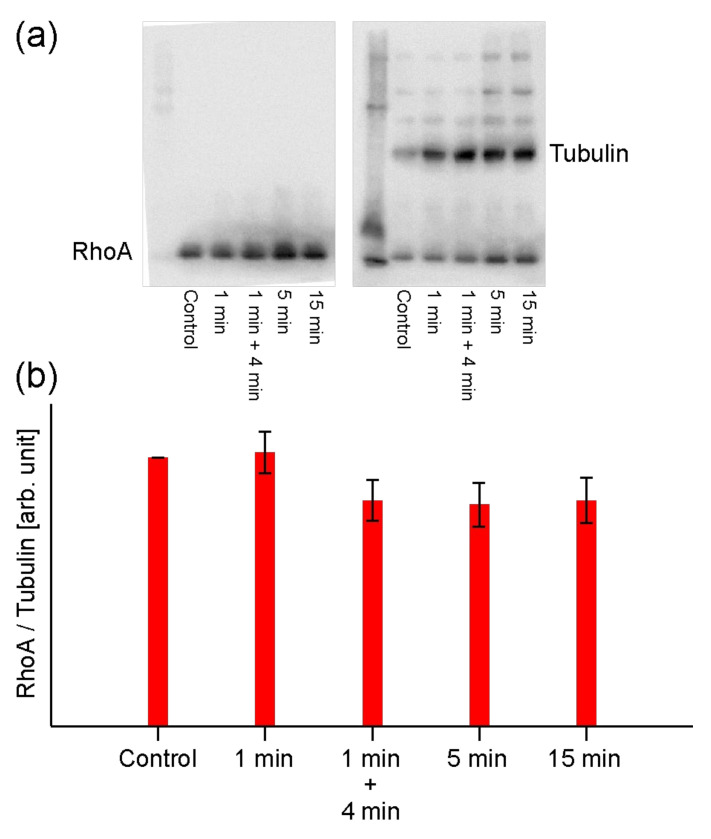
Results of estimating changes in RhoA concentration after PDT with PLP by Western blotting. (**a**) Western blot photograph. (**b**) Graph quantifying the results of (**a**). The irradiation was carried out at 0.0531 mW/cm^2^.

Figure 5 shows the results. In the PDT with PLP, there was no change in the concentration of RhoA after 1 min of irradiation, and the amount of RhoA decreased when the cells were incubated for 4 min at 37 °C in 5% CO_2_ after 1 min irradiation. The decrease in RhoA after 5 min and 15 min irradiation was almost the same as after 1 min of irradiation and 4 min of standing. Therefore, it is considered that the RhoA*/ROCK binding was saturated after 1 min of irradiation. In a previous experiment using NPe6, the concentration of RhoA decreased after 1 min of irradiation and RhoA*/ROCK was saturated after 5 min of irradiation [19].

The difference between PDT using NPe6 and PLP is the difference in the decrease in RhoA concentration (activation of RhoA to RhoA*) due to 1 min of irradiation, and it is considered that NPe6 forms RhoA*/ROCK more rapidly. However, the saturation of RhoA*/ROCK and the initiation of blebbing due to the overactivation of myosin light chains shown in Figure 2 are faster for PLP. This indicates that the timing at which ROS is generated and RhoA is activated upon irradiation with light is different for NPe6 and PLP. That is, NPe6 gradually generates ROS that promotes RhoA activation immediately after light irradiation and forms RhoA*/ROCK more slowly than PLP. However, although PLP generates singlet oxygen (^1^O_2_) immediately after light irradiation, it does not generate ROS that promotes RhoA activation.

### 2.5. Elastic Modulus Measurement by AFM

**Figure 6:**
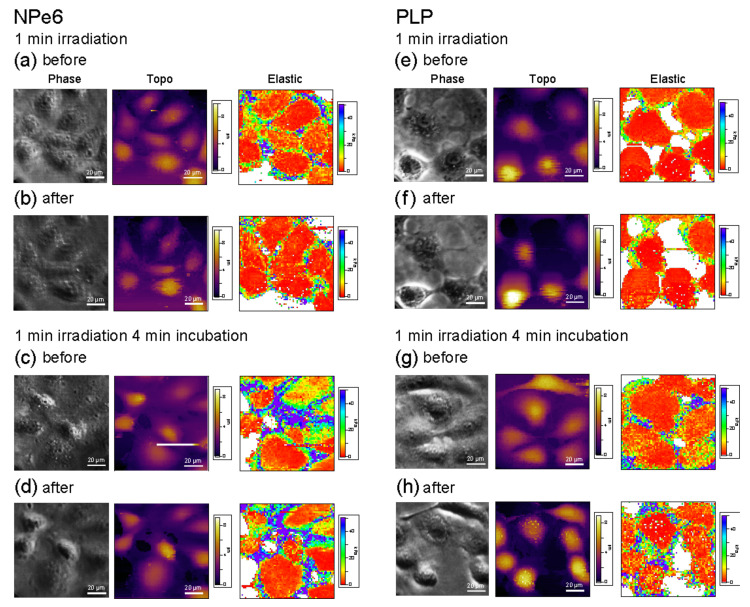
Phase-contrast images, topographic images, and elastic modulus maps of RGK1 with added NPe6 (**a**–**d**) and PLP (**e**–**h**) after 1 min of light irradiation and 4 min of standing in an incubator at 37 °C with 5% CO_2_ concentration. Force curves were measured at 64 × 64 points in an area of 100 μm × 100 μm. The topo images were obtained by mapping the height when a force of 200 pN was measured. The elastic modulus was evaluated using Hertz’s formula [38].

To examine the above results using changes in cell stiffness, we measured the local elastic modulus of cells using AFM. Since the state of actin filament formation is related to the elastic modulus of the cell, it is possible to determine the state of actin filament formation by measuring the elastic modulus of the cell [19]. Figure 6 shows phase-contrast images, topographic images, and elastic modulus maps of RGK1 with added NPe6 and PLP after 1 min of light irradiation and 1 min irradiation and 4 min standing in an incubator at 37 °C with 5% CO_2_ concentration.

**Figure 7:**
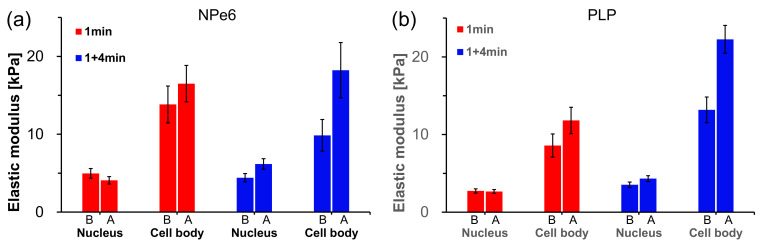
Estimated average local elastic moduli around the cell nucleus and the edge of the cell (referred to cell body) over all observed cells. N = 44 (1 min) and 45 (1 min + 4 min) for NPe6 (**a**), and 34 (1 min), and 26 (1 min + 4 min) for PLP (**b**), respectively.

For individual cells, we estimated the average local elastic modulus around the cell nucleus and the edge of the cell (referred to as the cell body). We also estimated the average elastic modulus over the observed cells, and the results are shown in Figure 7.

For NPe6, the elastic modulus showed little change after 1 min of light irradiation. After 1 min of irradiation and 4 min of standing in an incubator, the elastic modulus increased at the cell body and around the cell nucleus. This result corresponds well to the formation of stress fibers, as observed in a previous study [19]. In contrast, for PLP, the elastic modulus near the cell nucleus did not change after 1 min of light irradiation, but the elastic modulus at the cell body increased slightly. When the elastic modulus changes as a result of a change in osmotic pressure due to cell contraction, the elastic modulus of the cell increases uniformly and independently of the cell nucleus periphery and the cell body. Therefore, it is considered that this change in elastic modulus reflects some change in the cell structure. After 1 min of irradiation and 4 min of standing in an incubator, the elastic modulus of the edge of the cell increased remarkably, approaching the state of NPe6. This corresponds well to the results for actin filament generation shown in Figure 4. However, from the phase-contrast images in Figure 2, the cells contracted for PLP under both 1 min of light irradiation and 1 min of irradiation followed by 4 min of standing in a incubator, whereas the contraction of cells was negligible for NPe6. These results suggest the existence of a new mechanism for the case of PLP-PDT, which we will discuss in detail later.

For NPe6, the average elastic moduli around the cell nucleus and at the edge of the cell were estimated to be 4.97 ± 0.63 kPa and 13.8 ± 2.4 kPa before light irradiation and 4.08 ± 0.48 kPa and 16.5 ± 2.3 kPa after 1 min irradiation, respectively. The average elastic modulus around the cell nucleus did not change markedly, and the average elastic modulus at the cell body increased by 19% (1.19-fold increase). The average elastic moduli around the cell nucleus and at the cell body were estimated to be 4.41 ± 0.54 kPa and 9.86 ± 2.03 kPa before light irradiation, which increased to 6.17 ± 0.68 kPa and 18.2 ± 3.6 kPa after 1 min of light irradiation and 4 min of standing, respectively. The average elastic modulus of the edge of the cell increased by 85% (1.85-fold increase). The delay of 5 min for stress fiber formation is related to the activation of myosin light chain after light irradiation [19,32].

For PLP, the average elastic moduli around the cell nucleus and at the edge of the cell were estimated to be 2.74 ± 0.27 kPa and 8.58 ± 1.49 kPa before light irradiation and 2.67 ± 0.24 kPa and 11.8 ± 1.7 kPa after 1 min of light irradiation, respectively. The average elastic modulus around the cell nucleus did not increase, but the average elastic modulus at the cell body increased by 38% (1.38-fold increase), which is a larger increase than for NPe6. The average moduli of elasticity around the cell nucleus and at cell body was estimated to be 3.53 ± 0.35 kPa and 13.2 ± 1.7 kPa before light irradiation, which increased to 4.33 ± 0.37 kPa and 22.3 ± 1.8 kPa after 1 min of light irradiation and 4 min of standing in the incubator, respectively. The average moduli of elasticity around the cell nucleus and the cell body increased. However, the rate of increase in the average elastic modulus of the edge of the cell was large, increasing by 69% (1.69-fold increase).

The standard error of the average elastic modulus after 1 min of irradiation and 4 min of standing was smaller for PLP than for NPe6. This suggests that PLP has a more uniform effect on the whole cell, which may be consistent with the high therapeutic efficacy of PDT observed in in vivo studies [18].

### 2.6. Mechanistic Differences between NPe6 and PLP

Based on the above results, we consider the reason for the difference in the effects of NPe6 and PLP on PDT. NPe6 and PLP absorb the light around 655 nm. The molecular structure that generates singlet oxygen is a porphyrin ring in NPe6 and a porphyrin ring and chlorine in PLP [13,14,15,18]. We discuss why the timing and amount of RhoA activation and switching of the RhoA*/ROCK generation mechanism are different.

**Figure 8:**
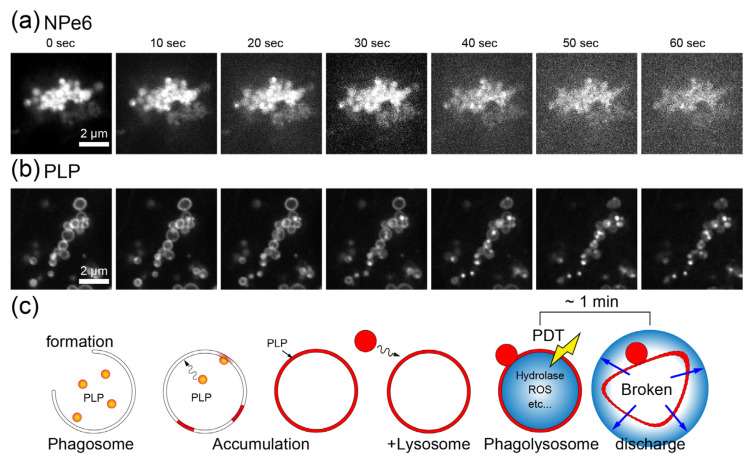
Confocal fluorescence observation images of (**a**) NPe6 and (**b**) PLP during 1 min of irradiation with light of 640 nm wavelength and 439 mW/cm^2^. (**c**) Schematic diagram of PLP uptake and destruction of phagosome membrane. In (**a**), the contrast has been adjusted.

Figure 8a,b, respectively show confocal fluorescence observation images of NPe6 and PLP added samples during 1 min of irradiation with light of 640 nm wavelength at 439 mW/cm^2^. It has been suggested that NPe6 is taken up by lysosomes and PLP by phagosomes. For NPe6, no significant structural changes were observed in the lysosomes where NPe6 is thought to accumulate. In contrast, phagosomes are deformed after irradiation, and almost all of them are destroyed within 50 s to 1 min.

Figure 8c shows a schematic diagram of PLP uptake by phagosomes and the destruction of the phagosome membrane by PDT. PLP induces phagosome formation and is incorporated therein. PLP is considered to accumulate in the phagosome membrane, and thereby a ring-like structure was observed in the fluorescence image shown in Figure 1. Phagosomes and lysosomes are known to fuse to form phagolysosomes [23,26,27,28,29,30,31]. As shown in Figure 1, phagosomes are fused with granular substances with a diameter of several hundred nanometers that emit strong fluorescence, which could be lysosomes. Phagolysosomes are known to produce non-mitochondrial generation of ROS [39]. Therefore, in addition to ROS generated from the porphyrin ring and chlorine of PLP, other ROS are also generated by the oxidation of substances flowing from lysosomes. It is considered that large amounts of ROS are released when the phagosome membrane is destroyed by light irradiation of the PLP accumulated there. The reason why RhoA is not immediately activated by light irradiation in PLP, unlike NPe6, is well explained by the fact that it takes time for the phagolysosomal membrane to be destroyed.

As clarified in previous NPe6 research, there is a threshold amount of ROS involved in the increase in actin filament formation [19]. That is, there is an upper limit to the concentration of RhoA that is activated and binds to ROCK. When an amount of ROS that greatly exceeds this threshold is generated in a short time, the surplus ROS destroy cells regardless of the increase in actin filament formation [19]. When the amount of ROS generated exceeds the threshold but does not cause cell necrosis, the concentration of RhoA, which binds to ROCK, reaches the upper limit and the elastic modulus stops increasing. This is because the amount of ROCK is almost fixed. After 1 min of irradiation and 4 min of standing in an incubator, the amount of singlet oxygen generated is greater in PLP than in NPe6. However, as shown in Figure 7, both PLP and NPe6 resulted in similar increases in the average elastic modulus of the cell edges. This is probably because both photosensitizers reached the upper limit of the concentration of RhoA* that binds to ROCK.

Finally, we consider the reason why the elastic modulus of the cell edge slightly increased immediately after irradiation in the case of PLP. As shown in the schematic diagram of Figure 8c, the phagosome membrane is broken, and the release of ROS and hydrolytic enzymes causes cell contraction, increasing the tension at the cell edges and the local elastic modulus. However, this observation is inconsistent with the absence of RhoA activation as shown in Figure 5. On the other hand, it is known that actin polymerization and pseudopodia formation are induced during phagosome formation [29]. Therefore, the increase in the elastic modulus of the cell edges at the early stage of PDT with PLP may be caused by such a mechanism, which is different from the mechanism activating RhoA with ROS discussed in the case of NPe6. Nevertheless, it was revealed that PLP induces RhoA activation through a mechanism different from that of NPe6 and exhibits different dynamical effects of PDT.

## 3. Materials and Methods

NPe6 is a photosensitizer with a porphyrin ring and has been widely used in PDT. PLP is a photosensitizer that contains a hydrophobic drug-loadable core enveloped in a porphyrin–lipid monolayer and constrained by ApoA-1 mimetic R4F (Ac-FAEKFKEAVKDYFAKFWD) peptide networks [14,15]. The cells used in this study were rat gastric mucosa-derived cancer-like mutant RGK1 cells [20]. RGK1, a chemically induced oncogenic cancer-like mutant of RGM1 (RGM1, a rat gastric epithelial cell line, was purchased from RIKEN CELLBANK (Ibaraki, Japan)), was established [20]. RGK1 cells were cultured in DMEM/F12 without L-glutamine (Sigma-Aldrich Japan K.K., Tokyo, Japan). NPe6 (final concentration 78 μM) or PLP (final concentration 19 μM) added to samples, which were allowed to stand for 24 h in an incubator at 37 °C with a CO_2_ concentration of 5%. These values are not toxic [18]. In this study, it was carried out for 24 h so that the amount of sensitizer entering the cells was saturated. There is an experiment in which the incubation time was varied [18]. It has been confirmed that it does not saturate in 3 h but saturates in 12 h. We used 24 h to saturate the uptake. Phase-contrast and fluorescence observations and their time-lapse observations were performed with a IX83 microscope system (Olympus Corp.) equipped with a stage top incubator (Tokai Hit Co., Ltd.). A super-resolution observation unit (Yokogawa Electric Corp., CSU-W1 SoRa) was installed in the IX83 system and used to observe the accumulation sites of NPe6 and PLP. For the light irradiation of PDT, a white light source of the IX83 system for fluorescence observation equipped with a 655 nm filter and a 640 nm semiconductor laser in the CSU-W1 SoRa system was used.

NPe6 or PLP was added to RGK1 cells cultured in a dish of 60 mm diameter and allowed to stand for 24 h in an incubator at 37 °C with a CO_2_ concentration of 5%. These cells were also used for Western blotting to quantify the amount of RhoA. AFM observation was performed by setting the AFM system (Oxford Instruments MFP-3D-BIO) on a microscope (Olympus Corp. IX71) so that the elastic modulus of the same cells could be measured before and after light irradiation. After AFM observation using the IX71 system, the sample was moved to the stage top incubator placed on an IX83, irradiated with light, and then returned to the IX71 system. The same cells were found by phase-contrast observation, and AFM observation was performed on them.

## 4. Conclusions

We investigated the difference in the mechanism of PDT between two photosensitizers, PLP, which has recently attracted attention for its potential to be highly effective in treating cancer, and NPe6, which has been widely used. It was revealed that PLP induces RhoA activation through a mechanism different from that of NPe6 and exhibits different dynamical effects of PDT: (1) NPe6 accumulates in lysosomes, whereas PLP is incorporated into phagosomes, whose formation is induced by PLP injection into cells. (2) PDT causes NPe6 to generate ROS, thereby producing actin filaments and stress fibers. (3) In the case of PLP, ROS generated by PDT remain in the phagosomes until the phagosomal membrane is destroyed, which delays the initiation of RhoA activation and RhoA*/ROCK generation. (4) However, after the disruption of the phagosome membrane, various ROS outflow, accelerating the production of actin filaments and stress fibers, and blebbing occurs earlier than in the case of NPe6. (5) In the initial stage of PDT, PLP increased the elastic modulus of cells without RhoA activity. The increase in the cell edge’s elastic modulus of the cell edges is caused by a mechanism different from that considered so far for NPe6. That may be because phagosomes are involved in polymerizing actin filaments and pseudopodia formation.

Considering the high selectivity and uptake of PLP into cancer cells, a greater effect with PDT can be expected by skillfully combining the newly discovered characteristics, such as the appearance of a strong effect at an early stage. For example, as mentioned in the introduction, it has been confirmed that PLP is highly effective against NOZ cells [18]. Considering that the mechanism is common, this result is expected to work as a similar mechanism for other cells. Since PLP accumulates in phagosomes, which can be visualized, it is also expected to play an important role in studying the details of phagosome (autophagosome) dynamics in living cells.

## Data Availability

Not applicable.
